# Expanded Transsphenoidal Trans-Lamina Terminalis Approach to Tumors Extending Into the Third Ventricle: Technique Notes and a Single Institute Experience

**DOI:** 10.3389/fonc.2021.761281

**Published:** 2021-12-08

**Authors:** Lei Cao, Wentao Wu, Jie Kang, Hui Qiao, Xiaocui Yang, Jiwei Bai, Haibo Zhu, Yazhuo Zhang, Songbai Gui

**Affiliations:** ^1^ Department of Neurosurgery, Beijing Tiantan Hospital, Capital Medical University, Beijing, China; ^2^ Beijing Neurosurgical Institute, Capital Medical University, Beijing, China

**Keywords:** trans-lamina terminalis approach, craniopharyngioma, third ventricle, chiasm pituitary corridor, expanded transsphenoidal

## Abstract

**Object:**

The trans lamina terminalis approach (TLTA) has been described as a way to remove third ventricular tumors. The aim of this paper was to analyze the feasible outcomes of TLTA applied to tumors extending into the third ventricle in our institute.

**Methods:**

Suprasellar tumors (n = 149) were treated by the extended endonasal approach from September 2019 to December 2020 in Beijing Tiantan Hospital. Eleven of the tumors were treated by TLTA or TLTA *via* the trans-chiasm-pituitary corridor (TCPC). The surgical technique notes of TLTA were described and indications and outcomes of the approach were analyzed.

**Results:**

There were 11 patients enrolled in the study, six with papillary craniopharyngiomas, two with adamantinomatous craniopharyngiomas, one with a germinal cell tumor (GCT), one with cavernous malformation and one with chordoid glioma. Four of the patients received a radical resection by TLTA alone, while seven of them received TLTA *via* the TCPC. Gross total resection was achieved in eight patients (72.7%), and partial resection in three patients (27.3%). Visual function was improved in four of the 11 patients (36.4%), was unchanged in five patients (45.5%), and deteriorated in two patients (18.2%). New-onset hypopituitarism occurred in seven patients (63.3%) and new-onset diabetes insipidus occurred in two patients (18.2%). Electrocyte imbalance were observed in six patients (54.5%) at post-operative week 2. There were no surgery-related deaths or cerebrospinal fluid leaks. Postoperative intracranial infection was observed in one patient (9.1%), and during the follow-up period, tumor recurrence occurred in one patient (9.1%).

**Conclusion:**

The expanded TLTA provides a feasible suprachiasm corridor to remove tumors extending into the third ventricle, especially for craniopharyngiomas. Sound understanding of the major strengths and limitations of this approach, as well as strategies for complication avoidance, is necessary for its safe and effective application.

## Introduction

Tumors located in the third ventricle behind the chiasma, such as craniopharyngiomas, are technically challenging due to their proximity to vital neurovascular structures including the hypothalamus, optic apparatus and anterior cerebral artery (ACA) complex. Access to these tumors is very difficult due to their deep location. King (1) described a trans-lamina terminalis approach (TLTA) through pterional craniotomy as a safe corridor to access these third ventricular lesions, as well as some other access options such as the transcallosal interforniceal approach (2, 3) and transcortical transforaminal approach (4). Compared to other approaches, TLTA provides direct access to the retro-chiasmatic portion of the tumor with little optic nerve retraction.

Expanded endonasal approaches (EEA) (5–7) can provide direct access to the midline skull base, including access to suprasellar tumors. However, reports on how to resect tumors involving the third ventricle have been limited. Kitano (8) reported extended transsphenoidal surgery for 20 suprasellar craniopharyngiomas using infrachiasmatic access, combined with or without a suprachiasmatic trans-lamina terminalis approach. Seo (9) also reported a series of 82 cases of tumors involving the third ventricle resected by EEA. Most of these cases were treated *via* the infrachiasm corridor or chiasm-pituitary corridor. However, resection of the tumor in the third ventricle *via* TLTA by extended transsphenoidal approach was seldom reported. In the present study, we share our experience with resection of tumors located in the third ventricle by TLTA in our single institute. The surgical technique notes and outcomes of TLTA are discussed as well as its advantages and disadvantages.

## Methods

### Patient Selection

A total of 149 patients with suprasellar tumors underwent surgery using EEA between September 2019 and December 2020 in the Department of Neurosurgery, Beijing Tiantan Hospital, Capital Medical University, by G.S.B. Eleven of the 149 tumors extended behind the chiasma into the third ventricle and were removed *via* TLTA with or without access through the trans-chiasm-pituitary corridor (TCPC) by EEA. This study was performed under an institutional review board-approved protocol in compliance with regulations set by our institution for the study of human subjects with their informed consent and was approved by ethics committee of Beijing Tiantan Hospital, Capital Medical University (KY 2021-041-02).

### Preoperative MRI Evaluation

**Figure 1:**
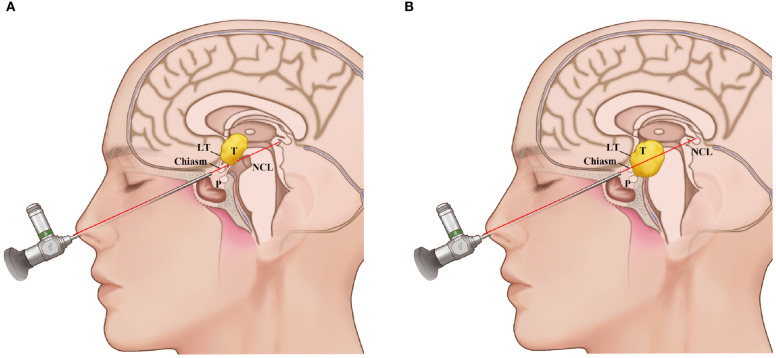
Schematic representation of the use of the nasal-chiasmic line for corridor selection in third ventricle lesion. **(A)**, If the percent of the supra-NCL region included over 20% of the tumor, then the lamina terminalis corridor was used to remove this part of the tumor. **(B)** If the area of the tumor in the infra-NCL region was over 10% and the area of the tumor in the infra-NCL region was over 20%, both lamina terminalis corridor and infrachiasmic corridor were used. NCL, nasal-chiasmic line; T, tumor; P, pituitary; LT, laminal terminalis.

All patients preoperatively received enhanced MRI examinations. All tumors were located retrochiasm in the third ventricle. A line between the nasal apex and the chiasm on the sagittal MRI (nasal-chiasm line, NCL) was used to evaluate the supra- and infrachiasm corridors available for tumor resection. The NCL divided the tumors into two areas, the upper supra-NCL region and the lower infra-NCL region. If the percent of the supra-NCL region included over 20% of the tumor, then the lamina terminalis corridor was used to remove this part of the tumor. At the same time, if the area of the tumor in the infra-NCL region was over 10%, both TLTA and TCPC were used (**Figure 1**).

### Surgical Techniques

All 11 patients underwent tumor resection using the expanded transsphenoidal approach. An intraoperative visual evoked potential (VEP) monitor was used to monitor visual function. According to the preoperative MRI evaluation, if TLTA could be used to access the supra-NCL part of tumor, then bone of the bilateral optic canal was fully removed to increase optic nerve mobilization during optic nerve retraction so as to reduce damage to the nerve. The dura mater was opened to fully expose both the suprachiasm and infrachiasm corridors. The arachnoid membrane surrounding the chiasm was dissected sufficiently to release the optic nerves. When dissecting the suprachiasmatic space, we took care to protect the anterior communicating artery complex and its branches to the optic chiasm. We then opened the lamina terminalis with scissors to expose the tumor in the third ventricle. First, the tumor was decompressed piece by piece patiently to gain sufficient surgical space. Then the extracapsular portion of the tumor was dissected away from the optic chiasm, hypothalamus and surrounding artery system, especially the posterior cerebral artery, *via* careful microdissection between the tumor capsule and arachnoid plane, and the tumor was removed piece by piece until finally achieving complete resection. For tumors with more than 10% of their area under the NCL, after the upper tumor was removed, the degree of optic chiasm mobilization increased significantly. The infrachiasmatic corridor then widened significantly to expose the tumor. Sometimes, when the bottom of the third ventricle was intact, it was necessary to open the capsule from the weakest part of the tumor surface and resect the residual tumor. It was also necessary to protect the pituitary stalk, superior pituitary artery, circle of Willis and its branches. Finally, the tumor was completely resected. The skull base reconstruction was performed according to our previous work (10). These technique notes were showed in Video 1 for Case 9.

All patients received intraoperative VEP monitoring to predict visual outcomes, as referred to in our previous work (11). More attention was given to VEP variations during the three stages of unroofing the optic canal, removing the tumor and reconstructing the skull base.

### Extent of Tumor Resection

The extent of resection was determined by pre- and postoperative volumetric analysis of MR images. Gross total resection (GTR) was defined as 100% tumor removal, subtotal total resection (STR) was defined as tumor removal of over 90%, and partial total resection (PTR) was defined as tumor removal less than 90%, but greater than 50%. Tumor recurrence during the follow-up period was defined as the appearance of new pathological tissue on MR images or the growth of tumor remnants. Follow-up MR imaging was performed at 3 months after surgery and then at regular intervals of 6–12 months.

### Visual Function

Visual acuity and visual field examinations were evaluated by an ophthalmologist before and after surgery.

### Endocrine Status

The endocrine status of all patients was assessed pre- and postoperatively according to adenohypophysis function and diabetes insipidus. Adenohypophysis function was assessed using complete serum pituitary hormone panels. Diabetes insipidus was defined as urine volume greater than 50 ml/kg/d. Electrocyte imbalance was defined as serum sodium level over 145 mmol/L or lower than 135 mmol/L, and serum potassium level over 5.5 mmol/L or lower than 3.5 mmol/L 2 weeks after surgery.

All patients BMIs were assessed at time of surgery and at the last visit. Obesity was defined as BMI > 30 kg/m^2^ or 9% BMI gain after surgery compared with the preoperative BMI (12).

## Results

### Demographic and Clinical Factors

**Figure 2:**
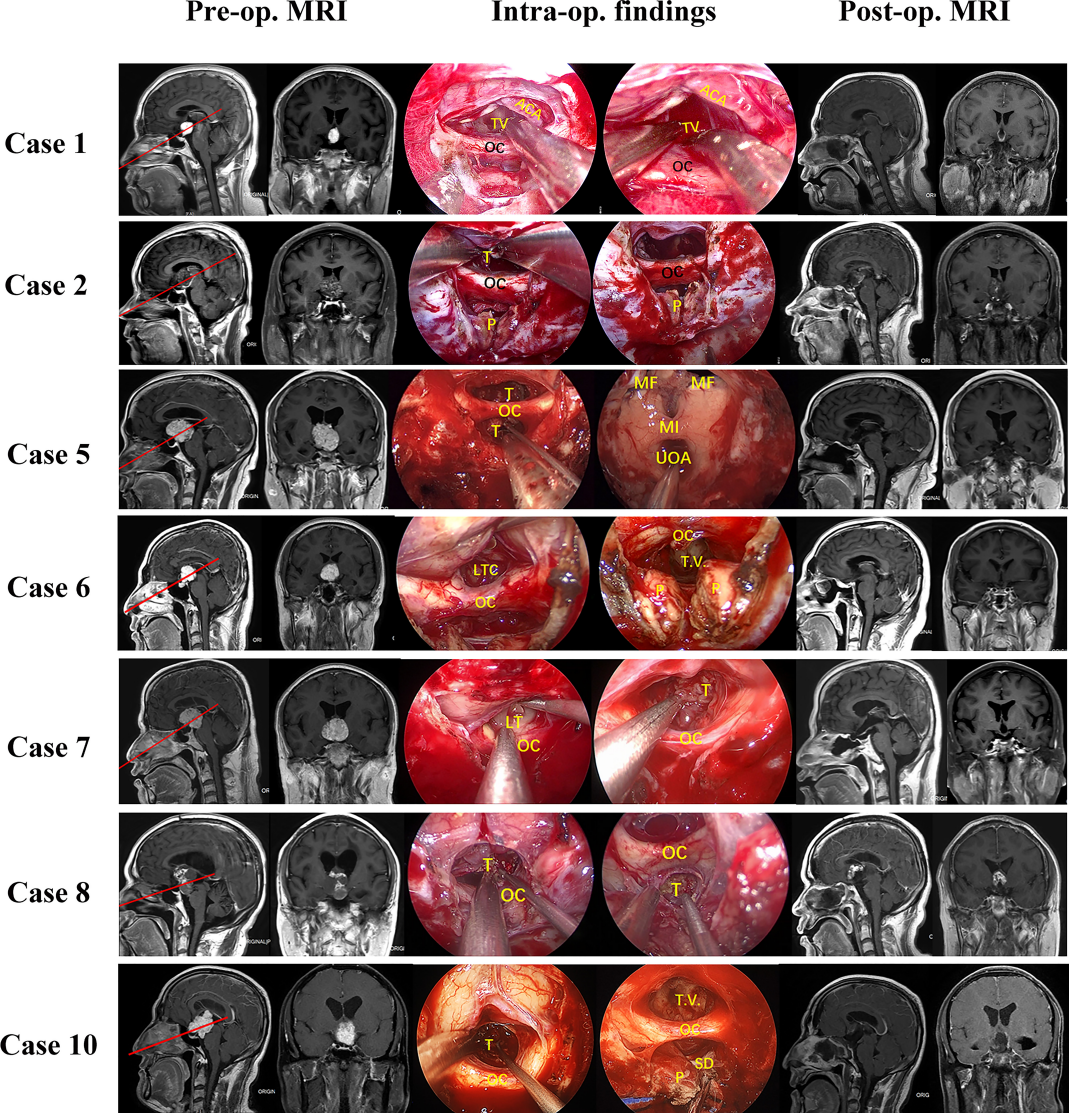
Pre- and post-operative MRI and intraoperative findings of 11 patients treat by trans-lamina terminalis approach combined with trans-pituitary-chiasm corridors. Case 1, a papillary craniopharyngioma located in the 3^rd^ ventricle with supra-NCL part about 50%. Removing the tumor through EEA with two corridors: suprachiasm trans-lamina terminalis corridor and infrachiasm chiasm-pituitary corridor. The tumor was gross total removed and lateral wall of third ventricle can be seen clearly. Case 2, a cavernous malformation located in the 3^rd^ ventricle with supra-NCL part about 80%. the tumor was solid with a large draining vein. Both TLTA and TCPC were used to remove the tumor. the tumor was partial removed for protection of the draining vein. Case 5, a papillary craniopharyngioma extended into the 3^rd^ ventricle with supra-NCL part about 95%. Removing the tumor through EEA with supra-chiasm corridor (trans-lamina terminalis). After the tumor was totally removed, the posterior wall of third ventricle, Monro foramen, and upper outlet of aqueduct can be seen clearly. Case 6, an ACP extended into the 3^rd^ ventricle with supra-NCL part about 70%. Both TLTA and TCPC were used to remove the tumor totally. Case 7, a papillary craniopharyngioma with supra-NCL part about70% were removed by both TLTA and TCPC. Case 8, an ACP with supra-NCL part about 55% were removed by both TLTA and TCPC. the tumor was adhered internal cerebral vein tightly and encased AComA complex, the superior part of tumor was left for gamma knife. Case 10, a papillary craniopharyngioma with supra-NCL part about 55% were removed totally by TLTA combined with TCPC. Case 11, a papillary craniopharyngioma with supra-NCL part about 90% were removed totally by TLTA. OC, optic chiasm; PS, pituitary stalk; ON, optic nerve; T, tumor; P, pituitary; ICA, internal carotid artery; T.V., 3^rd^ ventricle; LT, lamina terminalis; HT, hypothalamus; MF, Monro’s foramen; TLTA,trans-lamina terminalis approach; TCPC,trans pituitary-chiasm corridor.

**Figure 3:**
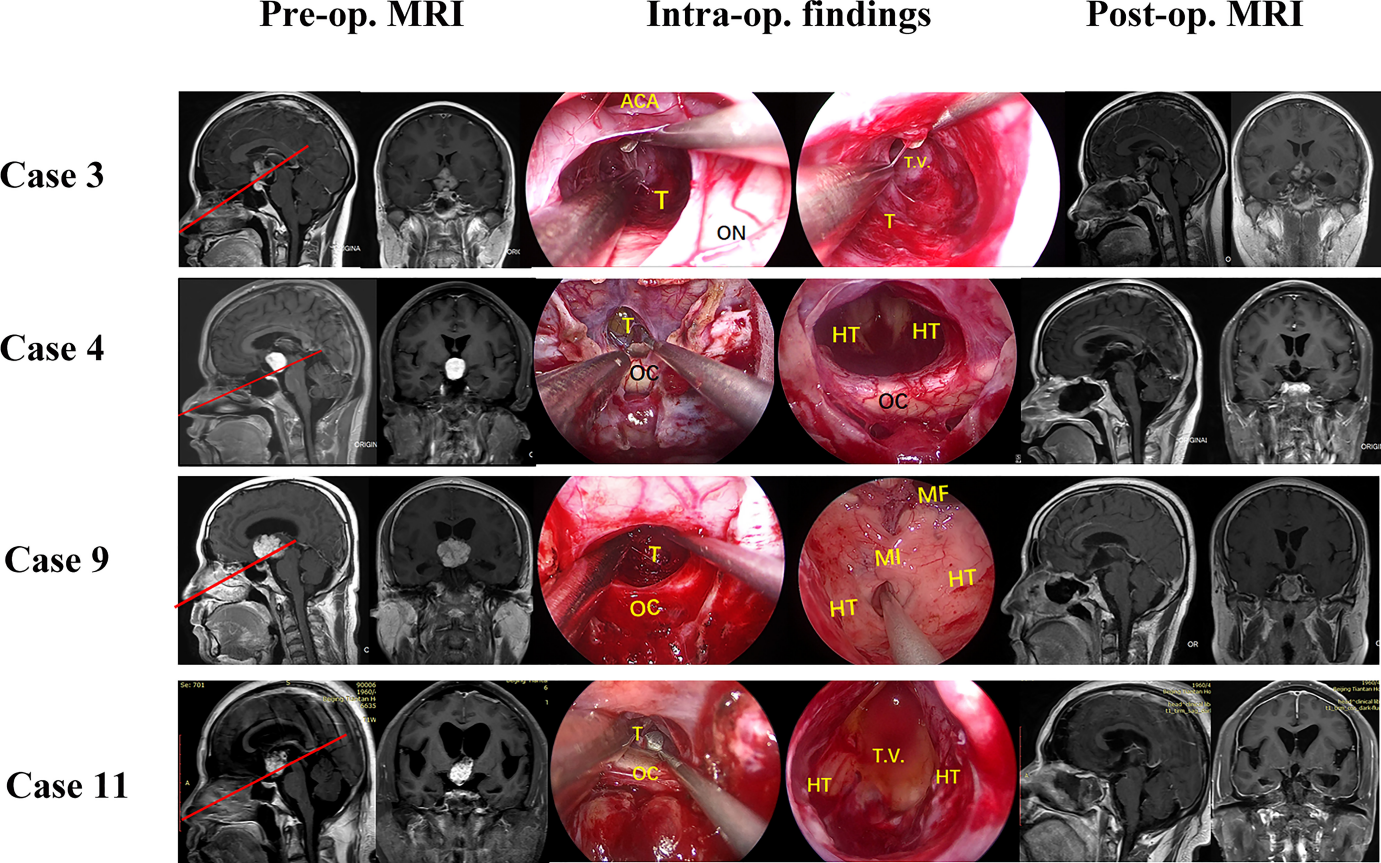
Pre- and post-operative MRI and intraoperative findings of 11 patients treat by combined trans-lamina terminalis and trans-pituitary-chiasm corridors. Case 3, a germinoma with supra-NCL part about 80% were partial resected *via* a TLTA and a radiotherapy and chemotherapy were followed. Case 4, a chordoid glioma with supra-NCL part about 95% were total removed by TLTA. Case 9, a papillary craniopharyngioma with supra-NCL part about 95% were removed totally by TLTA. Case 11, a papillary craniopharyngioma with supra-NCL part about 90% were removed totally by TLTA. OC, optic chiasm; PS, pituitary stalk; ON, optic nerve; T, tumor; P, pituitary; ICA, internal carotid artery; T.V., 3^rd^ ventricle; LT, lamina terminalis; HT, hypothalamus; MF, Monro’s foramen; TLTA,trans-lamina terminalis approach.

From September 2019 to December 2021, 11 patients (ten males, one female) with tumors extending into the third ventricle, which were resected by TLTA participated in this study (details in **Table 1**). The average patient age was 45.1 ± 11.5 years, ranging from 25–68 years. In total, seven patients (63.6%) had vision impairment preoperatively. Additionally, three patients (27.3%) had polydipsia or polyuria, and one patient suffered from epilepsy. According to the histological characteristics, they were six papillary craniopharyngiomas, two adamantinomatous craniopharyngiomas, one cavernous malformation, one germinal cell tumor (GCT) and one chordoid glioma. Based on the NCL, tumors extending more than 50% supra-NCL were observed in all 11 patients, while tumors extending more than 10% infra-NCL were observed in eight patients. Intraoperatively, TLTA was used alone in 4 patients (**Figure 2**) while the TCPC was combined with TLTA in 7 patients (**Figure 3**). In a patient with an infra-NCL tumor of over 20%, only TLTA was used as the surgical strategy to achieve partial resection, followed by radiotherapy and chemotherapy; in this case, the frozen histological report indicated a GCT (Case 3).

**Table 1:** Clinical data of tumors extended into third ventricle.

Case	Patho.	CTNNB1/BRAFV600E Mutation	Gender/age	symptoms	Approach	Supra-NCL/sub-NCL	Extent of resection	Pre-op.Visual deficit	Post-op.Visual deficit	Intra-op. VEP	Pre-op. hypopituitarism	Post-op. hypopituitarism	Pre-op. DI	Post-op. DI	Pre-op. BMI	Pre-op. BMI	Adjunctive therapy	Recurrent
1	PCP	-/+	M/40	Tongue numbness	TLTA+TCPC	50%/50%	GTR	Nor.	Nor.	No-change	Nor.	N	N	N	29.4	31.2	N	N
2`	CM	NA	M/43	epilepsy	TLTA+TCPC	80%/20%	PTR	Nor.	Nor.	No-change	Nor.	Y	N	Y	24.7	25.3	N	N
3	GCT	NA	F/25	Polydipsia,polyuria, visual deficit	TLTA	80%/20%	PTR	Visual deficit	Worse	Worse	Nor.	Y	Y	Y	26.7	24.7	R+C	N
4	CG	NA	M/37	Polydipsia, polyuria, visual deficit	TLTA	95%/5%	GTR	Visual deficit	Improved	Improved	Nor.	Y	Y	Y	26.7	28.9	N	N
5	PCP	-/+	M/43	visual deficit	TLTA+TCPC	50%/50%	GTR	Visual deficit	No-changed	No-changed	Nor.	Y	N	Y	24.3	24.7	R	Y
6	ACP	NA*	M/46	visual deficit	TLTA+TCPC	70%/30%	GTR	Visual deficit	improved	Improved	Nor.	Y	N	N	23.4	24.3	N	N
7	PCP	-/+	M/55	visual deficit	TLTA+TCPC	70%/30%	GTR	Visual deficit	No-changed	No-changed	Nor.	Y	N	N	26.8	24.9	N	N
8	ACP	+/-	M/52	visual deficit	TLTA+TCPC	55%/45%	PTR	Visual deficit	Worse	Worse	Nor.	Y	N	N	32.4	28.7	Y	N
9	PCP	-/+	M/68	polydipsia, polyuria, visual deficit	TLTA	95%/5%	GTR	Visual deficit	Improved	Improved	Nor.	N	Y	Y	27.2	29.4	N	N
10	PCP	-/+	M/42	visual deficit	TLTA+TCPC	55%/45%	GTR	Visual deficit	Improved	Improved	Nor.	N	N	N	29.4	18.5	N	N
11	PCP	-/+	M/57	Memory disorder, visual deficit	TLTA	90%/10%	GTR	Visual deficit	Improved	Improved	Nor.	N	N	N	26.1	26.4	N	N

Patho., Pathology; PCP, papillary craniopharyngioma; CM,cavernous malformation; CG, chordoid glioma; TLTA, translamina terminalis approach; TCPC, trans chiasm-pituitary corridor; GTR, gross total resection; PTR, partial total resection; GCT, germinal cell tumor; ACP, adamantinomatous craniopharyngioma; DI, diabetes insipidus; Nor, normal; Y, yes; N, No; R, radiotherapy; C, chemotherapy; NA, Not available; *, specimen was not enough for detection of CTNNB1 and BRAFV600E mutation.

### Extent of Tumor Resection

GTR was achieved in eight (72.7%) patients. Partial resection was achieved in three patients. One patient with GCT achieved partial resection as the surgical outcome, followed by radiotherapy and chemotherapy. Another case was a cavernous malformation in which partial resection was used to protect an adherent large draining vein. A third case was craniopharyngioma in which the internal cerebral vein adhered tightly and the ACA was encased.

### Ophthalmologic and Endocrine Outcomes

Prior to surgery, nine patients with tumors extending into the third ventricle had visual deficits. After surgery, visual improvement was observed in four patients (36.4%), no change was seen in five patients (45.5%), and some deterioration occurred in two patients (18.2%). The ophthalmologic outcomes were consistent with the intraoperative VEP amplitude improvement.

Seven of 11 patients (63.6%) developed new adenohypophysis deficits postoperatively (**Table 2**) and three patients were unchanged. For most patients, the diabetes inspidus symptoms did not change in eight patients and new-onset symptoms occurred in two patients. Only one patient developed hypothalamic obesity with BMI of 31.2 (**Table 2**).

**Table 2:** Clinical outcomes of 11 patients with tumor resection by TLTA.

	NO.	Percentage
Extent of tumor resection		
GTR	8	72.7%
PTR	3	27.3%
Visual outcome		
Improved	4	36.4%
Not changed	5	45.5%
Worse	2	18.2%
Adenohypophysis function		
Improved	1	9.1%
Not changed	3	27.3%
Worse	7	63.6%
Diabetes insipidus		
Improved	0	0
Not changed	9	81.2%
Worse(new-onset)	2	18.2%
Post-op BMI>30 or 9% more BMI gained		
Yes	1	9.1%
Not	10	90.9%
Complications		
CSF leaks	0	0
Infections	1	9.1%
Electrolyte imbalance (in post-op. 2w)	6	54.5%
Mortality	0	0
Recurrent	1	9.1%

### Postoperative Complications and Outcomes

No postoperative cerebral spinal fluid leakage occurred. No patients died. One patient (Case 7) suffered from meningitis and was cured by administration of vancomycin and meropenem. Post-operative electrocyte imbalance including hypo- or hypernatremia and hypo- or hyperkalemia was observed in six patients (60%) after 2 weeks.

The mean follow-up period of this study was 12.16 ± 3.40 months (range, 6–22 months). One patient (case 3) with GCT who underwent PTR was treated with gamma knife therapy. One patient (case 7) with tumor recurrence received adjunctive gamma knife therapy. No tumor recurrence was observed in other patients.

### Illustrative Case

**Figure 4:**
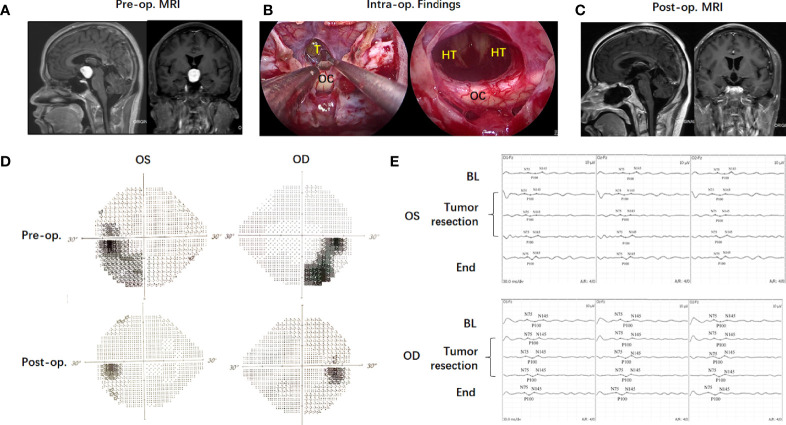
Case illustration of endoscopic transnasal translamina terminalis approach. A 37-year-old male patient (Case 4) with a solid lesion located in the third ventricle in preoperative MRI **(A)**. Based on the NCL, the Supra-NCL part was over 90%. Translamina terminalis approach was used to remove the tumor **(B)**. Postoperative MRI showed the tumor was totally removed **(C)**. The intraoperative VEP monitoring showed the VEP amplitude (N75 to P100) was gradually reversed and finally improved in both sides of eyes after the tumor was totally removed **(D)**. Postoperative ophthalmic examination showed the visual field improved significantly **(E)**.

A 37-year-old male patient (case 4) presented with polydipsia, polyuria and visual impairment for 1 year. Preoperative MRI indicated a solid lesion located in the third ventricle (**Figure 4**). Based on the NCL, the supra-NCL portion was over 90%. TLTA alone without TCPC was used to remove the tumor. Intraoperative VEP monitoring showed the VEP amplitude (N75 to P100) was gradually reversed and finally improved in both sides of the eyes after the tumor was totally removed. Postoperative ophthalmic examination showed that visual acuity and visual field improved significantly. The postoperative histological examination showed the tumor as a chordoid glioma and no tumor recurrence was observed 9 months post-operatively.

## Discussion

### Feasibility of Using TLTA for Tumors Extending Into the Third Ventricle by EEA

Tumors that are deep-seated mostly within the third ventricle are often considered for a midline approach such as endoscopic endonasal, subfrontal trans-lamina terminalis (13), anterior interhemispheric trans-lamina terminalis (14) or anterior transcallosal (15) approaches to gain optimal access and minimize hypothalamic disruption. EEA has become popular in recent decades to resect suprasellar tumor, but some authors do not support the use of EEA for tumors located in the third ventricle because the intact floor of the third ventricle may be at least partially violated by using the infrachiasmatic corridor (16). Recently, several authors have reported the outcomes of EEA for third ventricle tumors (17–21). In these studies, GTR ranged from 66.7%–90%, with newly developed endocrinopathy ranging from 18%–67% for panhypopituitarism, and the improvement rate for visual functions ranging from 56%–86%. The CSF leakage rate ranged from 3.8%–69%, with a recurrence rate ranging from 18%–34.4%. In our study, we report our successful experience in treating 11 patients with tumors located in the third ventricle by an expanded endoscopic transnasal approach *via* TLTA combined with or without access through TCPC. GTR was achieved in eight cases (72.7%). Visual impairments were improved or unchanged in nine cases (81.8%), with two new-onset diabetes inspidus, seven new onset cases of hypopituitarism, no surgical-related deaths and no CSF leaks. Only one patient had recurrent craniopharyngioma in 12 months. The results imply that it is feasible to remove deep-seated tumors in the third ventricle *via* TLTC by EEA.

The major finding of this report was that the patients achieved GTR after undergoing tumor resection *via* EEA even though a large amount of tumor extended up into the third ventricle above and behind the chiasm. Preoperatively, we evaluated the tumor topography using MRI. The suprachiasmatic and infrachiasmatic corridors were first evaluated with sagittal MRI. The NCL was used to divide the tumor into two-regions described as supra-NCL and infra-NCL. The surgical strategy was formulated in advance by evaluating the predominance of the supra-and infra-NCL regions. For tumors located in the third ventricle, the chiasm was likely to be compressed anterior-inferiorly, resulting in a chiasm-pituitary corridor too narrow to gain access to the tumor. For tumors with a supra-NCL region less than 20%, the chiasm was more likely to be compressed superiorly; thus, tumor resection could often be performed through the chiasm-pituitary corridor (22). With tumor decompression, the upper part of the tumor settled into the surgical field to be easily removed from the interface between tumor and hypothalamus. For tumors with a supra-NCL region more than 20%, the chiasms were more likely be compressed inferiorly, and the lamina terminalis provided good access to the upper part of the tumors. During the process of tumor depression, the chiasm resettled upward, so that TCPC was accessed to remove the lower part of the tumor. Therefore, the combination of TLTA *via* the TCPC was needed for this subtype of tumor. However, if the tumor region below the NCL was less than 10%, then the tumor could be removed by TLTA alone because the lower region could be moved upward by slight manipulation of the chiasm. For tumors in the third ventricle, intraoperative frozen pathology was needed to confirm the histological results. For germ cell tumors or optic gliomas, partial resection after tumor decompression was sufficient to reconstruct the cerebrospinal fluid circulation, and postoperative adjuvant radiotherapy and chemotherapy were then combined. For tumors with tight adhesion to important blood vessels such as the ACA or internal cerebral veins, a small amount of residual tumor was accepted if the tumor could not be separated, and adjuvant postoperative radiotherapy was employed to reduce tumor recurrence.

The limitation of TLTA is that higher surgical manipulation through the lamina terminalis may have a higher incidence of injury to the optic chiasm or anterior communicating artery (AComA) complex, because most of the blood supply to the optic apparatus comes from the branches of the ACA and AcomA (22, 23). Also, the AComA sometimes blocked the suprachiasmic corridors to expose the lesions in the third ventricle. So, some authors advocated the surgical clipping and division of the AComA in selected patients through the bifrontal basal interhemispheric approach or the anatomical feasibility through the endoscopic endonasal corridor to achieve a better visualization (24, 25). In our study, visual function worsened in 18.2% of the patients. We also used intra-operative VEP monitoring to assess manipulation of the optic nerve in order to avoid surgical damage to visual function (11). We also tried to remove more bone from the optical canals to increase the mobilization of the optic chiasm, which can reduce manipulation injuries though TLTA (26). The indications and complications of EEA TLTA for tumors extended into third ventricle were summarized in **Table 3**.

**Table 3:** The indications and complications of EEA TLTA for tumors extended into third ventricle.

	Details
Indications	All tumors were located retrochiasm in the third ventricle with the percent of supra-NCL part over 20 percent.
Complications	Visual impairment, endocrinological disorders, CSF leaks, electrocyte imbalance
Complications avoidance techniques	1.Protect the blood supply to chiasm, intra-operative VEP monitoring, reduce retraction of optic chiasm, remove the bone of optical canals to increase the optic nerve mobilization. 2.Skull base reconstruction using nasal septal flap. 3.Monitor the water and electrocyte balance.

### Comparison of Different Approaches to Tumors Extending Into the Third Ventricle

Currently, transcranial approaches such as the transcallosal interforniceal approach, transventricular approach and trans-lamina terminalis approach are commonly used to remove tumors extending into the third ventricle. The indications and pros and cons of these approaches are summarized in **Table 4**.

**Table 4:** Indications and pros and cons of different surgical approaches to the tumors extended to third ventricle.

Surgical approaches	Indications	Advantages	Disadvantages
Transcallosal interforniceal approach	Tumors favor the middle line and posterosuperior aspect of the third ventricles	Better for larger tumors and tumors that favor the posterosuperior aspect of the third ventricle Excellent window for the dissection of tumor from the hypothalamus bilaterally	Long work distance Cognitive impairment Limited visualization of the third ventricle floor and displaced optic chiasm from this superior viewpoint
Transventricular approach	Tumors favor the posterosuperior aspect of the third ventricles with dilated Monro’s Foramen or tumors extended into the lateral ventricle	1. Excellent visualization for larger tumors and tumors that favor the posterosuperior aspect of the third ventricle	Risk to the ipsilateral fornix and deep venous structures Long work distance Limited visualization of deep margin of the tumor
Anterior interhemispheric approach *via* TLTA	Tumors occupying the anteroinferior portion of the third ventricle without ACoA complex blocking lamina terminalis	Excellent visualization of tumors through lamina terminalis	Risks to the adjacent optic pathways, supraoptic nuclei of the hypothalamus, and columns of the fornix ACoA complex block TLTA
Subfrontal or pterion approach *via* TLTA	Tumors occupying the anteroinferior portion of the third ventricle without ACoA complex blocking lamina terminalis	Excellent visualization of contralateral wall of hypothalamus and third ventricle floor thought lamina terminalis	Limited visualization of the superior extent of tumor extension and ipsilateral wall of hypothalamus ACoA complex and ipsilateral A1 block TLTA
EEA *via* TCPC	Tumors favors sub-NCL part of the third ventricle	Excellent visualization of the infrachiasmic tumor little traction of optic chiasm	Long work distance Bad visualization of upper tumor to roof of third ventricle TCPC is narrow when chiasm prefixed CSF leaks
EEA *via* TLTA	Tumors favor supra-NCL part of the third ventricle without extending into lateral ventricle	Excellent visualization of the suprachiasmic tumor	1.High risk to optic chiasm for traction Limited visualization of infrachiasmic tumors Long wok distance CSF leaks

TLTA, translamina terminalis approach; TCPC, trans chiasm-pituitary corridor; EEA, extended endoscopic approach; ACoA, artery communicating artery; CSF, cerebrospinal fluid.

The transcallosal interforniceal route provides an excellent window for the dissection of tumors from the hypothalamus bilaterally, especially for larger tumors and tumors that favor the posterosuperior aspect of the third ventricle. However, direct visualization of the third ventricle floor and displaced optic chiasm from this superior viewpoint is unsatisfactory. Thus, tumor tissue in the anterior third ventricle and suprasellar cistern can be difficult to dissect safely given the working angle and depth, and resection can cause inadvertent damage of the third ventricle floor involving infundibulum/stalk disconnection and surrounding arteries. Furthermore, the transcallosal approach may cause cognitive decline in adults (27, 28).

In the transventricular approach, access into the third ventricle is performed *via* enlargement of the Monro foramen. However, the Monro foramen is more likely to be small in the absence of a dilated ventricular system, which will increase the risk to the ipsilateral fornix and deep venous structures. Additionally, the long working depth and blind spots in the transventricular approach can also complicate efforts to dissect the deep margin of the tumor from neurovascular structures when adherence is present.

The trans-lamina terminalis approach is suggested for tumors occupying the anteroinferior portion of the third ventricle. Entry through the lamina terminalis is associated with risks to the adjacent optic pathways, supraoptic nuclei of the hypothalamus and columns of the fornix. The small trans-lamina terminalis corridor is biased toward the inferior aspect of the third ventricle. There are various approaches for access to the lamina terminalis, such as the transcranial and transnasal approaches. When approached from the pterional corridor, lateral subfrontal and/or midline subfrontal interhemispheric corridor, limited visualization of the superior extent of tumor extension makes it difficult to reach tumors that extend posteriorly and superiorly. Elevation of the ACA also limits the superior trajectory of the exposure. Generally, the interface between the tumor and hypothalamus cannot be directly visualized through transcranial approaches, so it is often necessary to remove the tumor by traction. The damage to the hypothalamus caused by tumor traction is far greater than that caused by sharp dissection under direct vision. Therefore, if the tumor is integrated into the walls of the hypothalamus, many surgeons advocate forgoing total resection, leaving the part that adheres to the hypothalamus to avoid postoperative functional complications.

EEA has improved our ability to perform a cleaner dissection of the tumor away from the hypothalamus, with direct visualization of the interface between the tumor and hypothalamus. This can decrease damage to the hypothalamus caused by tumor traction, which means that EEA may be worthwhile to achieve GTR of the tumor with less damage to the hypothalamus (29, 30). Furthermore, radiotherapy to treat these tumors can also be damaging. In addition, the risk of re-operation for recurrent tumors after radiotherapy is higher, and the total resection rate can be significantly lower (31–33), especially for large cystic craniopharyngiomas, which tend to adhere more tightly to the hypothalamus, and from which a small residue may quickly grow into a large cyst tumor. Therefore, we attempt total resection whenever possible by careful sharp dissection between the tumor and hypothalamus to avoid these postoperative complications.

## Conclusions

TLTA provides a feasible suprachiasmatic corridor to remove lesions extending into the third ventricle by EEA. Sound understanding of the major strengths and limitations of this approach, as well as strategies for complication avoidance, is necessary for its safe and effective application.

## Data Availability Statement

The raw data supporting the conclusions of this article will be made available by the authors, without undue reservation.

## Ethics Statement

The studies involving human participants were reviewed and approved by Ethic committee of Beijing Tiantan Hospital, Capital Medical University (KY 2021-041-02). The patients/participants provided their written informed consent to participate in this study.

## Author Contributions

Conception and design: SG, LC, and YZ. Surgical Intervene: LC, JK, HZ, and JB. Data Collection and Analysis: LC, WW, and JK. Technique support: HQ and XY. Drafting the article: LC and SG. Critically revising the article: all authors. Approved the final version of the manuscript on behalf of all authors: SG. Study supervision: all authors. All authors contributed to the article and approved the submitted version.

## Funding

This study was supported by the Capital’s Funds for Health Improvement and Research (grant no.2020-4-1077), the Beijing Municipal Science & Technology Commission (Z19110700660000) and Beijing Hospitals Authority Clinical Medicine Development of Special Funding Support (XMLX202108).

## Conflict of Interest

The authors declare that the research was conducted in the absence of any commercial or financial relationships that could be construed as a potential conflict of interest.

## Publisher’s Note

All claims expressed in this article are solely those of the authors and do not necessarily represent those of their affiliated organizations, or those of the publisher, the editors and the reviewers. Any product that may be evaluated in this article, or claim that may be made by its manufacturer, is not guaranteed or endorsed by the publisher.
